# Gonorrhea vaginitis in a pediatric patient: a case report

**DOI:** 10.11604/pamj.2021.38.358.28390

**Published:** 2021-04-14

**Authors:** Ayu Wulansari Bambang, Idrianti Idrus, Safruddin Amin, Muji Iswanty

**Affiliations:** 1 Department of Dermatology and Venereology, Faculty of Medicine, Hasanuddin University, Makassar, Indonesia

**Keywords:** Gonorrhea vaginitis, prepubertal child, case report

## Abstract

Gonorrhea is all diseases caused by Neisseria gonorrhoeae. Prepubertal child is more susceptible to N. gonorrhoeae infection because the vagina is alkaline and contains no estrogen. Gonorrhea vaginitis is the most common form of gonorrhoea in prepubertal children beyond neonatal period. Transmission in child can be through sexual contact (abuse) or non-sexual contact. Gonorrhea vaginitis in children more often asymptomatic, with clinical manifestation such as mucopurulent discharge, vaginal pruritus and vulval erythema. Supporting examination comprise of gram staining from vaginal discharge, culture and nucleic acid amplification testing (NAAT). Ceftriaxone is drug of choice gonorrhea without complication in children. We report a case of 4 year and 9-month female girl that was diagnosed by history taking and supporting examination from gram staining and polymerase chain reaction (PCR) from vaginal discharge, and then treated with single dose ceftriaxone 125 mg intramuscular that gave clinical improvement.

## Introduction

Gonorrhea caused by *Neisseria gonorrhoeae* [1]. Humans are the only natural hosts for gonococci and can only last a short time outside the human body. Gonococci can infect mucous membranes predominantly covered by columnar epithelial cells, namely the urethra, cervix, rectum, pharynx, conjunctiva and vagina of prepubertal women [2]. Gonorrhea vaginitis is the most common form of gonorrhea in children beyond the neonatal period. Data on the prevalence of gonorrhea infection in prepubertal children and young adolescents are limited. The incidence rate of gonorrhea in the 1 month - 9-year age group of 11 per 100,000 has been reported by a Florida surveillance data. There is no screening in the pediatric age group and diagnosis based solely on tests performed on symptomatic patients. Recognizing gonorrhea in children after the neonatal period and before puberty is very important because it is an indicator of sexual harassment/violence. However, non-sexual transmission of gonorrhea has been reported in several studies and case reports [3,4]. Gonorrhea vaginitis in children is often asymptomatic in 15-44% cases. The most common manifestation is a purulent vaginal discharge with large amounts of white, yellow to greenish color which can stain the underwear. Itching, dysuria and an erythematous vulva may also appear [5]. Gram stain is inadequate for evaluating gonorrhea in prepubertal children and cannot be used to diagnose or rule out a diagnosis of gonorrhea [6]. Nucleic Acid Amplification Testing (NAAT) includes polymerase chain reaction (PCR) can be used for examination of specimens from the vagina or urine [6,7]. We reported a case of gonococcal vaginitis in a 4 years and 9 months old child based on history, gram and PCR examination of vaginal swab and treated with a single dose of 125mg ceftriaxone intramuscularly.

## Patient and observation

**Figure 1:**
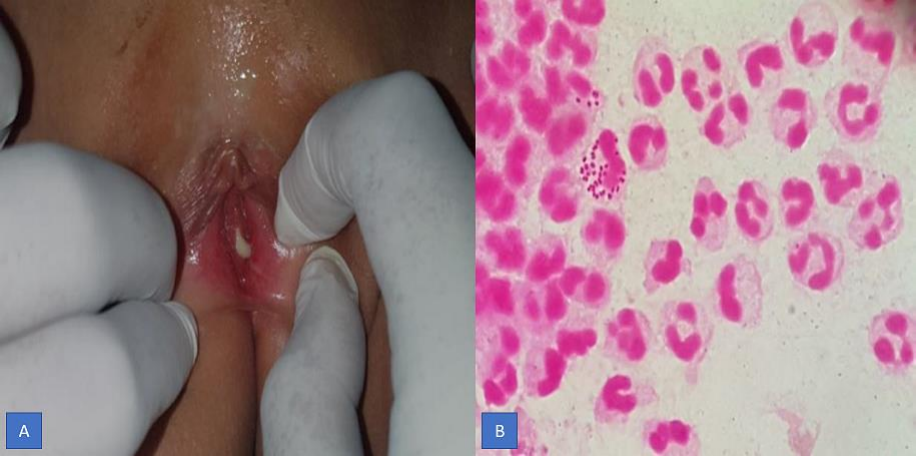
A) vulval redness and greenish yellow discharge; B) gram stain shows intracellular diplococci

**Figure 2:**
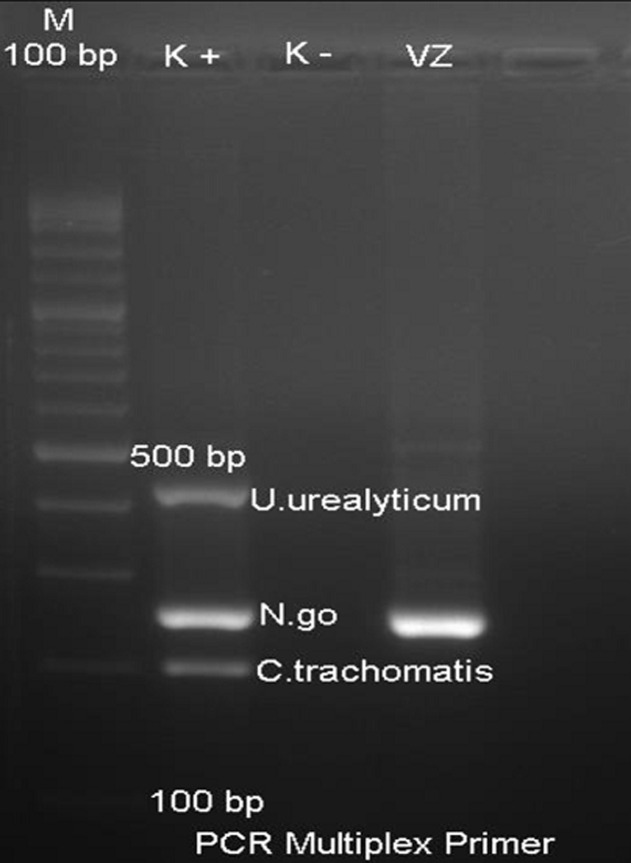
primary multiplex PCR examination showed *N. gonorrhoeae*

**Figure 3:**
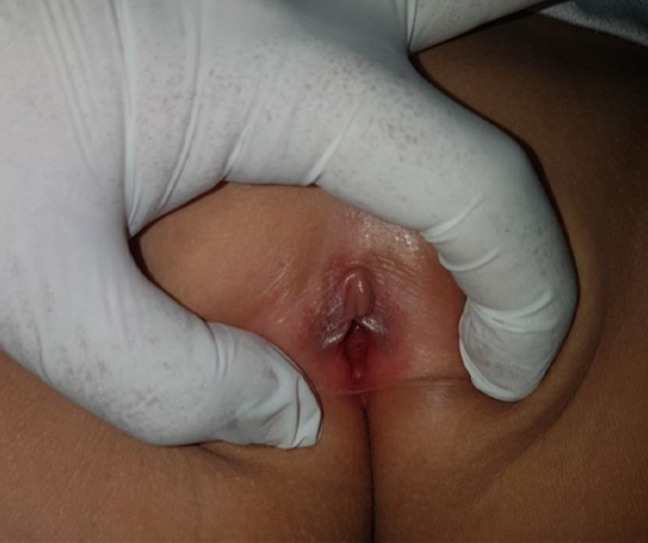
no more discharge was found after seven days of treatment

A 4 years and 9 months girl came to Hasanuddin University Hospital, South Sulawesi, Makassar, with a complaint of large quantities greenish yellow discharge from the vagina for 2 weeks. Vaginal discharge was associated with itch sensation at pubic area. She denied any fishy odor, dysuria, and fever. There was no change in behavior and the sufferer was always under close supervision from his mother. Her father was also complained of a penile discharge. Previous treatment history was amoxicillin 3x250mg for 3 days followed by metronidazole 3x250mg for 1 week without any improvement. General physical examination was remarkable. Venereological examination of the vulva and vaginal introitus showed vulvar erythema and greenish-yellow body discharge with negative whiff test (Figure 1A). Genital examination showed intact hymen with no signs of violence. Investigation in the form of examination of vaginal secretions with gram stain showed intracellular and extracellular gram-negative diplococci, with polymorphonuclear (PMN) leukocytes >25/LPB (Figure 1B). The PCR examination of vaginal secretions identified *Neisseria gonorrhoeae* (Figure 2). From the history, physical examination and investigations, the diagnosis of gonococcal vaginitis was confirmed. Injection of ceftriaxone 125mg intramuscularly was given. One week after therapy, the patient reported the vaginal discharge and the pruritus had subsided. The vulva was not an erythematous vulva and no discharge was found (Figure 3).

## Discussion

Gonococcal vaginitis is the most common form of gonorrhea in children beyond the neonatal period. Vaginal mucosa of prepubertal children is more susceptible to gonococcal infection due to its lower estrogen level which results in thinner mucosa in comparison to adolescent and adult. Prepubertal children also have an alkaline vaginal pH (6.5-7.5) which makes them susceptible to gonococcal infection and colonization. In addition, prepubertal children has lack of protective vulval fat pads and pubic hair, labia minora tend to be more open when children squat, thinner, smoother and more sensitive vulvar skin. Vaginal environment in prepubertal children acts as a good culture medium because it is warm, moist with a relatively normal pH to alkaline, thinner vaginal mucosal cell layers, atrophy due to low levels of the hormone estrogen, and poor personal hygiene in children [5]. Clinical symptoms that arise are usually in the form of a large amount of mucopurulent discharge that are white, yellowish to greenish in color which can stain the underwear. Discharge can also be present in small amounts. Vaginal itching, vulvar erythema and dysuria may also be present. Symptoms usually occur for 1 week, but in some cases, symptoms can last 2 weeks to months before finally being taken for examination. Gonorrhea is less common in boys [8].

The adhesion of the gonococcus is preceded by a pili structure whose receptors are only found in humans. The previously suspected pili receptor was CD46, which has now changed. Gonococci also use the Opa protein and possibly the lipooligopilisaccharide (LOS) to bind strongly to host cells during cellular invasion. There are multiple forms of Opa protein and each Opa binds to various receptors. Other gonococcus adhesins include porin proteins that bind to CR3 receptors on female genital tract cells, and LOS, which bind to asialogycoprotein receptors on epithelial cells. The gonococci will then replicate intracellularly in the phagosomes and then exit through the basal cells to the submucosa via exocytosis. Progressive breakdown of mucosal cells and invasion of the submucosa is accompanied by a strong PMN leukocyte response, formation of submucosal microabscess formation, and exudation of purulent material into the lumen of the infected organ [1,3].

In this case, patient's father experienced the same symptom of penile whitish discharge. It is very important to recognize gonorrhea in prepubertal children beyond the neonatal period because it is an indicator of sexual violence. However, several studies and case reports have reported non-sexual transmission of gonorrhea. Gonococci can breed optimally at 25°-39°C and die at 55°C for 5 minutes, die quickly in natural environments and are susceptible to dry conditions, with an incubation period of 2-7 days. However, gonococcus have been found to persist on saline-dampened linen cloths for 5 hours to even 22 hours. The gonorrhea placed on a slide can last up to 24 hours and on a towel for up to 17 hours at room temperature [4]. Numerous studies have been conducted to determine the survival time of gonococcus on some materials that may be contaminated by infected individual. Gonococci are found in almost all materials after 24-48 hours and even certain materials are found to survive up to 72 hours or more. In this case the suspicion of sexual abuse in children can be ruled out due to intact hymen and no signs of violence. Patients also did not experience behavioral change [9].

In this case, investigations were performed using gram stain and PCR of vaginal secretions. Gram staining revealed intra and extracellular diplococci with PMN> 25/LPB. PCR results with primers of *N. gonorrhoeae, Chlamydia trachomatis* and *Ureaplasma urealyticum* have identified *N. gonorrhoeae*. From the literature, it is stated that the microbiological diagnosis of gonorrhea in children must be carried out using a technique that has high specificity and sensitivity. This is due to the high social response to this disease, as well as for medical purposes and its relationship with sexual abuse in children [1]. Gram stain is inadequate for evaluating gonorrhea in prepubertal children, and cannot be used to diagnose or rule out a diagnosis of gonorrhea., because of the possibility of infection by another Neisseria strain. Gonococcal culture is a sensitive and specific test and is also inexpensive. However, it is considered less than ideal for routine diagnostics because of the stringent requirements for specimen collection and transport and confirmation takes some time. However, the advantage of gonococcal culture is that it can be used for antimicrobial susceptibility testing in culture isolates and also for genetic analysis if needed. NAAT is considered to have high specificity and sensitivity so that it can be used as an alternative culture for examining specimens from the vagina or urine [9].

The patient was treated with a single dose of 125mg ceftriaxone. The CDC STD Treatment Guidelines 2015 recommend a ceftriaxone regimen of 25-50 mg/kg intravenously or intramuscularly in a single dose, maximal dose 125 mg, for infants and children weighing ≤ 45 kg in uncomplicated gonococcal vaginitis [8]. The pattern of gonococcus susceptibility is very dynamic and can vary greatly between areas. Currently, there is a worldwide prevalence of gonococcal resistance to antimicrobials of sulfonamides, penicillin, early generation cephalosporins, tetracyclines, macrolides, and fluoroquinolones. In most countries, the main choice for empiric therapy for gonorrhea is extended-spectrum cephalosporins (ESCs), oral cefixime, and particularly the more potent injection ceftriaxone [10].

## Conclusion

Gonorrhea infection should be considered in vaginal discharge. Sexual abuse should always be investigated in gonorrhea infection. Appropriate antibiotic associated with good prognosis. Early detection should be done to prevent further complication.
